# Cancer cell‐derived novel periostin isoform promotes invasion in head and neck squamous cell carcinoma

**DOI:** 10.1002/cam4.5601

**Published:** 2023-01-23

**Authors:** Shao Wenhua, Takaaki Tsunematsu, Masaaki Umeda, Hiroaki Tawara, Natsumi Fujiwara, Yasuhiro Mouri, Rieko Arakaki, Naozumi Ishimaru, Yasusei Kudo

**Affiliations:** ^1^ Department of Oral Bioscience Tokushima University Graduate School of Biomedical Sciences Tokushima Japan; ^2^ Department of Oral Molecular Pathology Tokushima University Graduate School of Biomedical Sciences Tokushima Japan; ^3^ Department of Oral Healthcare Promotion Tokushima University Graduate School of Biomedical Sciences Tokushima Japan

**Keywords:** head and neck squamous cell carcinoma, invasion–, isoform, metastasis, Periostin

## Abstract

It recently has been reported that partial‐epithelial–mesenchymal transition (p‐EMT) program is associated with metastasis in head and neck squamous cell carcinoma (HNSCC). We previously have identified *POSTN* (which encodes periostin) as an invasion‐promoting molecule in HNSCC. Interestingly, *POSTN* expression is frequently observed in cancer cells with higher p‐EMT score by using a previous single‐cell transcriptomic data of HNSCC cases. Although it is known that *POSTN* has 11 splicing variants, the role of them has not been determined in HNSCC. Here, we found that HNSCC cells with EMT features expressed *POSTN* isoforms, Iso3 (lacking exon 17 and 21) and Iso5 (lacking exon 17), whereas fibroblast expressed Iso3 and Iso4 (lacking exon 17, 18, and 21). The expression of *POSTN* Iso3 and Iso4 are known to be widely observed in various cell types including stromal cells. Therefore, we focused on the role of novel cancer cell‐derived *POSTN* isoform, Iso5, in HNSCC. Single overexpression of *POSTN* Iso5 as well as Iso3 promoted invasion. Surprisingly, Iso5 synergistically promoted invasion together with Iso3. Notably, Iso5 as well as Iso3 upregulated p‐EMT‐related genes. We suggest that a novel cancer‐specific *POSTN* isoform lacking exon 17 (Iso5) can be a useful marker for detecting cancer cells undergoing p‐EMT. Moreover, a *POSTN* Iso5 can be a novel target for diagnosis and therapy in HNSCC.

## Graphical abstract

In this study, we identified a POSTN isoform lacking exon 17 (Iso5) as a promoting factor of tumor progression via p‐EMT program in HNSCC. HNSCC cells having co‐expression of POSTN Iso3 and Iso5 may possess more aggressive invasive potential. We suggest that POSTN Iso5 can be a useful marker for detecting cancer cells undergoing EMT.

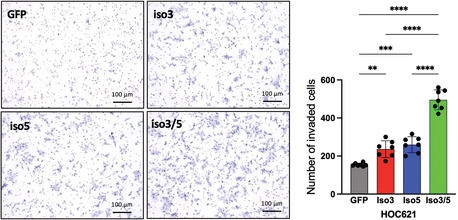

## 1 INTRODUCTION

Head and neck squamous cell carcinoma (HNSCC) has an annual incidence of over 500,000 cases worldwide.^1^ HNSCC has an approximately 50% of 5‐year survival rate. Unfortunately, in over 2 decades, the 5‐year survival rate has not improved.^2^ In a multistep process of HNSCC development, multiple genetic and epigenetic alterations are accumulated.^3^ We previously identified *POSTN* (which encodes periostin) by comparing the gene expression profiles of parent cells and their highly invasive clone.^4^ Periostin encoded by the *POSTN* gene (GenBank: D13664) is a secreted and a vitamin K‐dependent glutamate‐containing matricellular protein.^5^, ^6^ To date, *POSTN* expression has been shown to be induced by growth factors (Transforming growth factor‐β1 (TGF‐β1), TGF‐β2, TGF‐β3, BMP‐2, BMP‐4, Vascular Endothelial Growth Factor (VEGF), connective tissue growth factor 2 (CTGF2)), interleukins (IL‐3, −4, −6 and − 13), vitamin K, and valsartan in a cell‐specific context.^7^

In various cancers, the elevated *POSTN* expression is observed and is involved in malignant behaviors.^8^ We previously found that ectopic periostin promotes invasion, anchorage‐independent growth, angiogenesis, lymphangiogenesis, and metastasis.^4^, ^9^ Bao et al. demonstrated that periostin overexpression displayed accelerated metastatic growth of colon cancer cells and promoted cancer cell survival via activation of Akt/PKB pathway.^10^ Clinical studies in oral, breast, and colon cancers reveal that *POSTN* is well correlated with angiogenesis and metastasis.^9^, ^10^, ^11^, ^12^ In addition, periostin drives the epithelial–mesenchymal transition (EMT) via induction of matrix metalloproteinase 9 (MMP‐9), MMP‐10, and MMP‐13 expression, resulting in local tumor spread via extracellular matrix degradation.^13^, ^14^, ^15^ Furthermore, periostin plays a role in remodeling of the tumor microenvironment.^4^, ^16^, ^17^, ^18^, ^19^ On the other hand, metastatic colonization can be achieved by stromal periostin via the interaction between cancer stem cells and their metastatic niche.^20^, ^21^, ^22^

EMT is defined by a loss of epithelial cell junctions and polarity and an acquisition of mesenchymal properties.^23^ In early stage of metastatic process, cancer cells lose cell–cell adhesion and increase the mobility via EMT.^24^ During EMT induction during cancer progression, solid cancer cells exhibit the hybrid epithelial and mesenchymal characteristics, and this process is known as a partial EMT (p‐EMT).^25^, ^26^, ^27^, ^28^ It is thought that the p‐EMT program in cancer cells is involved in enhanced invasive properties, generation of circulating tumor cells, and resistance to anticancer drugs.^29^, ^30^ It is believed that p‐EMT rather than complete EMT possesses a higher metastatic risk. Single‐cell transcriptional profiles from HNSCC patients reveal that a p‐EMT program exhibits the expression of extracellular matrix (ECM) proteins and lacks the expression of classical EMT transcription factors.^31^

So far, 11 different splice variants of *POSTN* have been described.^7^, ^32^, ^33^ However, functional implications of *POSTN* splicing variants are not fully understood yet. Potentially, in various tissues and disease states, distinct splicing variants may have different functions. In this study, therefore, we investigated the expression patterns and the role of *POSTN* splicing variants of HNSCC cell lines. A novel cancer cell‐derived *POSTN* isoform (Iso5) was specifically identified in HNSCC cells and synergistically enhanced invasion with a common isoform (Iso3).

## 2 MATERIALS AND METHODS

### 2.1 scRNA‐seq data analysis

The processed single‐cell RNA‐seq data obtained from the GEO database (accession number: GSE103322) was used.^30^ The log‐transformed expression data from the cancer cells or noncancer cells were re‐analyzed using the *Seurat* (v4.1.1) R package.^31^ Clustering analysis was initially performed with default settings using all cells, including cancer or noncancer cells. We used top 15 principal components (PCs) as an input to Uniform Manifold Approximation and Projection (UMAP) analysis. The resolution parameter was set to 0.4 for the FindClusters function. Then, the eight cancer cells in the noncancer cell cluster were removed and the clustering analysis was performed again with cancer cells (PCs:13, resolution:0.6). The second‐level clustering for MEEI5/16 cluster was performed with the different parameter (resolution = 0.4) based on the re‐calculated top 10 PCs. Differential gene expression analysis between *POSTN* expressing and nonexpressing cells in MEEI16 tumor was performed with FindMarkers function and top20 differential‐expressed genes were visualized.

The p‐EMT scores for reported p‐EMT signatures^31^ were calculated by single sample gene enrichment analysis using the *ConsensusTME* R package.

### 2.2 TCGA data analysis

RNA‐seq data of 564 (520 tumors, 44 normal) head and neck cancers (TCGA‐HNSC) were obtained using *RTCGA* R package. The fibroblast scores in each TCGA samples were calculated by ssGSEA using the *ConsensusTME* R package. The three‐year overall survival of HNSC patients with higher and lower expression in POSTN were analyzed using *survival* and *survminer* R package.

### 2.3 Reagents and antibodies

Growth factors were obtained from the following companies: TGF‐β1 (#240‐B‐010/CF), HGF (#294‐HG‐005/CF), and PDGF (#220‐BB‐010, R&D Systems, Inc.); basic FGF2 (#064–04541, FUJIFILM Wako Pure Chemical Corp.); and EGF (#100–47, Pepro Tech). We generated a polyclonal anti‐periostin antibody for detection exon 21 by using specific peptides (GHLFEDEEIKRLLQGC). For recognizing all periostin isoforms, we used an anti‐periostin antibody against N‐terminal of periostin (ab14041, Abcam). A mouse monoclonal anti‐GFP antibody and a mouse monoclonal β‐actin antibody were obtained from Wako and Sigma, respectively.

### 2.4 Cell culture

HSC2, HSC3, HSC4, and MRC‐5 were obtained from JCRB (Japanese Collection of Research Bioresources Cell Bank). HOC313, HOC621, HOC719‐PE, and HOC719‐NE cells were obtained from Prof. Nobuyuki Kamata (Hiroshima University).^34^ These cells were maintained in Dulbecco's Modified Eagle's Medium (#044–29765, FUJIFILM Wako Pure Chemical Corp.) supplemented with heat‐inactivated 10% fetal bovine serum (#10099–141, Invitrogen) at 37°C in 5% CO_2_. MSCC‐Inv1 cells were established in our laboratory^4^ and were maintained in Keratinocyte‐SFM (#17005042, Invitrogen) under a condition of 5% CO_2_ in air at 37 °C.

### 2.5 RT‐PCR

By using the RNeasy Mini Kit (#74106, Qiagen), total RNA was isolated from cells. Their purity was determined by a standard spectrophotometric method. From 500 ng total RNA, cDNA was synthesized by using the PrimeScript RT Master Mix (#RR036A, Takara Bio Inc). We used the following primers. human *POSTN*: forward, 5′‐CTCATAGTCGTATCAGGGGTCG‐3′ and reverse, 5′‐ACACAGTCGTTTTCTG‐3′; human *GAPDH*: forward, 5′‐TCCACCACCCTGTTGCTGTA‐3′ and reverse, 5′‐ACCACAGTCCATGCCATCAC‐3′ (product size, 450 bp). For analyzing splicing variants, we used the following primers. F1: CTTCAAAGAAATCCCCGTGAC; F2: GGAGGTGGAGAAACAGAAGAAA, R1: TCTTCTGTTTCTCCACCTCCA; R2: CAACTTCCTCACGGGTGTGT. Total cDNA was amplified with GoTaq® Green Master Mix (#M712, Promega). PCR was performed by using a T100 thermal cycler (Bio‐rad) for 35 cycles after an initial 2 min denaturation at 95°C, annealed for 30 sec at 58°C for F1/R1, 56.5°C for F1/R2 and 57°C for F2/R2, and then extended for 1 min at 72°C in all primers. The PCR products were electrophoresed on 1.2% or 3.0% agarose/TAE gels (#02468–66, Nakalai tesque, Inc.) at 100 mV and visualized by ethidium‐bromide staining.

### 2.6 Quantitative RT‐PCR

*POSTN* mRNA expression was determined by using a LightCycler 96 system (Roche) with SYBR Premix Ex Taq II reagent (#RR820A, Takara Bio). For detecting *POSTN* or *GAPDH* expression, the following primers were used. *POSTN*: forward, 5′‐CTCATAGTCGTATCAGGGGTCG‐3′ and reverse, 5′‐ACACAGTCGTTTTCTG‐3′; *GAPDH*: forward, 5′‐TCCACCACCCTGTTGCTGTA‐3′ and reverse, 5′‐GCATCCTGGGCTACACTGAG‐3′. Relative *POSTN* mRNA expression was normalized by *GAPDH* mRNA.

For the expression of p‐EMT genes, the following primers were used. SERPINE1: forward, 5′‐cgctgggaaagca ttaagag‐3′ and reverse, 5′‐cacgcccagctaatttttgt‐3′; TGF Beta Induced (TGFBI): 5′‐gtgtgtgctgtgcagaaggt‐3′ and reverse, 5′‐ttgagagtggtagggctgct‐3′; MMP10: 5′‐ggctctttcactcagcc aac‐3′ and reverse, 5′‐tcccgaaggaacagattttg‐3; LAMC2: 5′‐gtcactggagaacgctgtga‐3′ and reverse, 5′‐agacccatttcgtt ggacag‐3′; P4HA2: 5′‐tgtcaaactgacaccccgta‐3′ and reverse, 5′‐ggactcctgtcttgggatca‐3′; PDPN: 5′‐catcgaggatctgccaac tt‐3′ and reverse, 5′‐acgatgattgcaccaatgaa‐3′; ITGA5: 5′‐ct acaatgatgtggccatcg‐3′ and reverse, 5′‐ggatatccattgccatcc ag‐3′; LAMA3: 5′‐agatgaggcacatggagacc‐3′ and reverse, 5′‐ttcttttgcgctttgtgttg‐3′; CDH13: 5′‐tgatgatgccaaaaacctca‐3′ and reverse, 5′‐atgggcaggttgtagtttgc‐3′; TNC: 5′‐ggtacagt gggacagcaggt‐3′ and reverse, 5′‐gttaacgccctgactgtggt‐3′; MMP2: 5′‐atgacagctgcaccactgag‐3′ and reverse, 5′‐atttg ttgcccaggaaagtg‐3′; EMP3: 5′‐gtggtctcagcccttcacat‐3′ and reverse, 5′‐atgagggagagcaccatgag‐3′; INHBA: 5′‐cctcgga gatcatcacgttt‐3′ and reverse, 5′‐ccctttaagcccacttcctc‐3′; LAMB3: 5′‐gggggagatcacaaacttga‐3′ and reverse, 5′‐gtg ctggcagacacagacat‐3′; VIM: 5′‐attgcaggaggagatgcttca‐3′ and reverse, 5′‐gtggagtttcttcaaaaaggca‐3′; THBS2: 5′‐tcctg aaaacaatgccatca‐3′ and reverse, 5′‐gtccacagacccaaactcgt‐3′; CXCL13: 5′‐ctctgcttctcatgctgctg‐3′ and reverse, 5′‐tgaggg tccacacacacaat‐3′; FN1: 5′‐cagtgggagacctcgagaag‐3′ and reverse, 5′‐gtccctcggaacatcagaaa‐3′; MMP3: 5′‐gcagtttgct cagcctatcc‐3′ and reverse, 5′‐gagtgtcggagtccagcttc‐3′; MMP9: 5′‐ttgacagcgacaagaagtgg‐3′ and reverse, 5′‐gccattcacgtcg tccttat‐3′; RAB25: 5′‐ccatcacctcggcgtactat‐3′ and reverse, 5′‐tttgttacccacgagcatga‐3′; MT1X: 5′‐accccaactgctcctgct‐3′ and reverse, 5′‐tctgacgtccctttgcagat‐3′; GPX3: 5′‐tgcaacc aatttggaaaaca‐3′ and reverse, 5′‐ttcatgggttcccagaagag‐3′; SPP1: 5′‐cccacagacccttccaagta‐3′ and reverse, 5′‐ggggacaa ctggagtgaaaa‐3′; MXD1: 5′‐tgaacatggttatgcctcca‐3′ and reverse, 5′‐acttgattcgggtccaagtg‐3′. Relative mRNA expression of p‐EMT genes was normalized to *GAPDH* mRNA.

### 2.7 Generation of periostin isoform 3‐ and 5‐overexpressing HNSCC cells

Lentiviral vector and packaging plasmids (psPAX2 and pMD2.G) were obtained from Addgene. Lentiviral vectors expressing GFP, periostin Iso3, and Iso5 were generated by using In Fusion cloning kit (#NC1845088, Clontech) from pEGFP‐C1 (#6084–1, Clontech) or pcDNA3.1‐periostin Iso3 and Iso5 obtained from Genscript. Lentiviruses were generated by co‐transfection with each amount of lentiviral vector and packaging plasmids into Lenti‐X 293 T cells (#NC9834960, Clontech) using PEI max reagent (#24765–100, Polyscience, Warrington, USA). After 48 hours of transfection, supernatants were collected and filtered using a 0.45 μm membrane. Filtered supernatants with 4 μg/mL polybrene directly infected to HOC621 cells. After 24 hours, the medium was changed by fresh media with 1 μg/mL of puromycin (#A1113803, Invitrogen).

### 2.8 Western blot analysis

Western blotting was performed as described previously.^4^ GFP, periostin Iso3‐ and Iso5‐overexpressing HOC621 cells were treated with 3 μM of monensin (#M5273, Sigma, Burlington, USA) for 24 hours. The intracellular protein expression of periostin is low because of the secretion from intracellular into extracellular. To quantify intracellular protein expression of periostin, we utilized monensin known as a protein transport inhibitor. Monensin blocks intracellular protein transport processes of any secreted proteins and induces the accumulation of these proteins in the Golgi complex. The increased accumulation of secreted proteins enhances the detectability of periostin with western blot analysis utilizing total cell lysate. Monensin treatment is well‐characterized method for detecting cytokines in intracellular staining with flowcytometry and immunofluorescence.^35^, ^36^ Then, cells were lysed using lysis buffer (50 mM pH 7.6 Tris–HCl, 150 mM NaCl, 1 mM EDTA, 1.5 mM MgCl_2_, 0.5% Nonidet P‐40, and 10% glycerol) with protease inhibitor cocktail (#04080‐24, Nacalai tesque). After centrifugation at 17,000 g for 20 min, the supernatant was collected. Protein concentration was measured using Bio‐Rad protein assay dye reagent (#5000006, Bio‐Rad laboratories Inc) by the absorption at 595 nm using a microplate reader (SpectraMax i3, Molecular Devices). Using 5%–20% gradient polyacrylamide gel (#E‐T520L, ATTO Corporation), 25 μg of protein were electrophoresed followed by blotting onto a nitrocellulose membrane (#10600093, Cytiva). By using an Immobilon HRP substrate (EMD Millipore Corporation), the signal was detected by a Fusion SOLO 7 S. EDGE (VILBER).

### 2.9 RNA Interference

Cells were seeded at a density of 1 × 10^5^ cells on a 6 cm dish and then transfected with oligonucleotides by using Lipofectamine RNAiMax (#100014472, Invitrogen). After 48 hours of transfection, total RNAs and lysates were prepared and analyzed by qPCR and immunoblotting, respectively. HOC719‐NE cells expressed periostin Iso3 and 5. To specifically knockdown *POSTN* Iso5, siRNAs were designed for targeting *POSTN* exon 21. For silencing all *POSTN* splicing variants, a 21‐bp duplex oligoribonucleotides corresponding to nucleotides 2397–2417 of the human *POSTN* mRNA sequence was used (Thermo Fisher Scientific). For silencing *POSTN* Iso 5, a siRNA targeting 5′‐GAAGAUGAAGAAAUUAAAAGA‐3′ (siRNA Ex21) was used (GeneDesign Inc.). The negative control siRNA was obtained from Horizon Discovery Ltd.

### 2.10 In vitro invasion assay

Invasion ability was determined by an in vitro invasion assay using an 8 μm pore cell culture insert (#3097, Becton Dickinson) in a 24‐well plate. Twenty micrograms of Matrigel (#354234, Becton Dickinson) was coated on the filter as a reconstituted basement membrane substance. Five hundred microliters of complete media or serum‐free medium (for TGF‐β1 treatment) were added to the lower compartment. 1.0–1.5 × 10^5^ cells were plated on the upper compartment of the cell culture insert with 100 μl of complete media or serum‐free medium. After incubation for 24 h at 37°C, the cells were fixed with formalin and stained with hematoxylin (#131‐09665, FUJIFILM Wako Pure Chemical Corp). By wiping with a cotton swab, the cells on the upper surface of the filter were removed, and the number of invaded cells (cells on the lower surface of the filter) was counted under a light microscope at 100x magnification. We assayed three times, and counted randomly selected three fields on the filter.

### 2.11 Statistical analysis

For qRT‐PCR analysis, the *t*‐test was used for comparing two groups. For in vitro invasion assay, data were processed in GraphPad Prism 9 (GraphPad) for statistical analyses using one‐way analysis of variance. For comparing p‐EMT and fibroblast scores and gene expression values between two groups in RNA‐seq data, Wilcoxon signed‐rank test was used. A *p*‐value of <0.05 was considered as a significant difference.

## 3 RESULTS

### 3.1 Expression of *POSTN* in HNSCC tissues

In previous studies, *POSTN* expression was observed in both cancer cells and stromal cells.^37^ To know *POSTN* expression in various cell types within the HNSCC tissue, we used the processed RNA‐seq data obtained from the GEO database with single‐cell transcriptomes from HNSCC patients.^31^ The log‐transformed expression data from the cancer cells or noncancer cells was re‐analyzed using the *Seurat* (v4.1.1) R package.^38^ Single‐cell profiles of malignant and nonmalignant cells highlighted the composition of the tumor microenvironment. By the expression pattern of known marker genes of stromal cells including fibroblasts, endothelial cells, muscle satellite cells (MuSC), T cells, B/plasma cells, macrophages, dendritic cells, and mast cells, the nonmalignant cells were partitioned to annotated clusters (Figure S1A). *POSTN* expression was mainly observed in endothelial cells and cancer‐associated fibroblasts (CAFs) (Figure S1B). By analyzing using bulk RNA‐seq data (TCGA‐HNSC), *POSTN* expression in tumor tissues was lower than that in normal tissues (Figure S1C). This may reflect the lower fibroblast abundance in cancer tissues (Figure S1D). Three‐year survival between patients with higher and lower POSTN expression shows no difference, which may be reflected by POSTN expression in noncancer cells.

**FIGURE 1:**
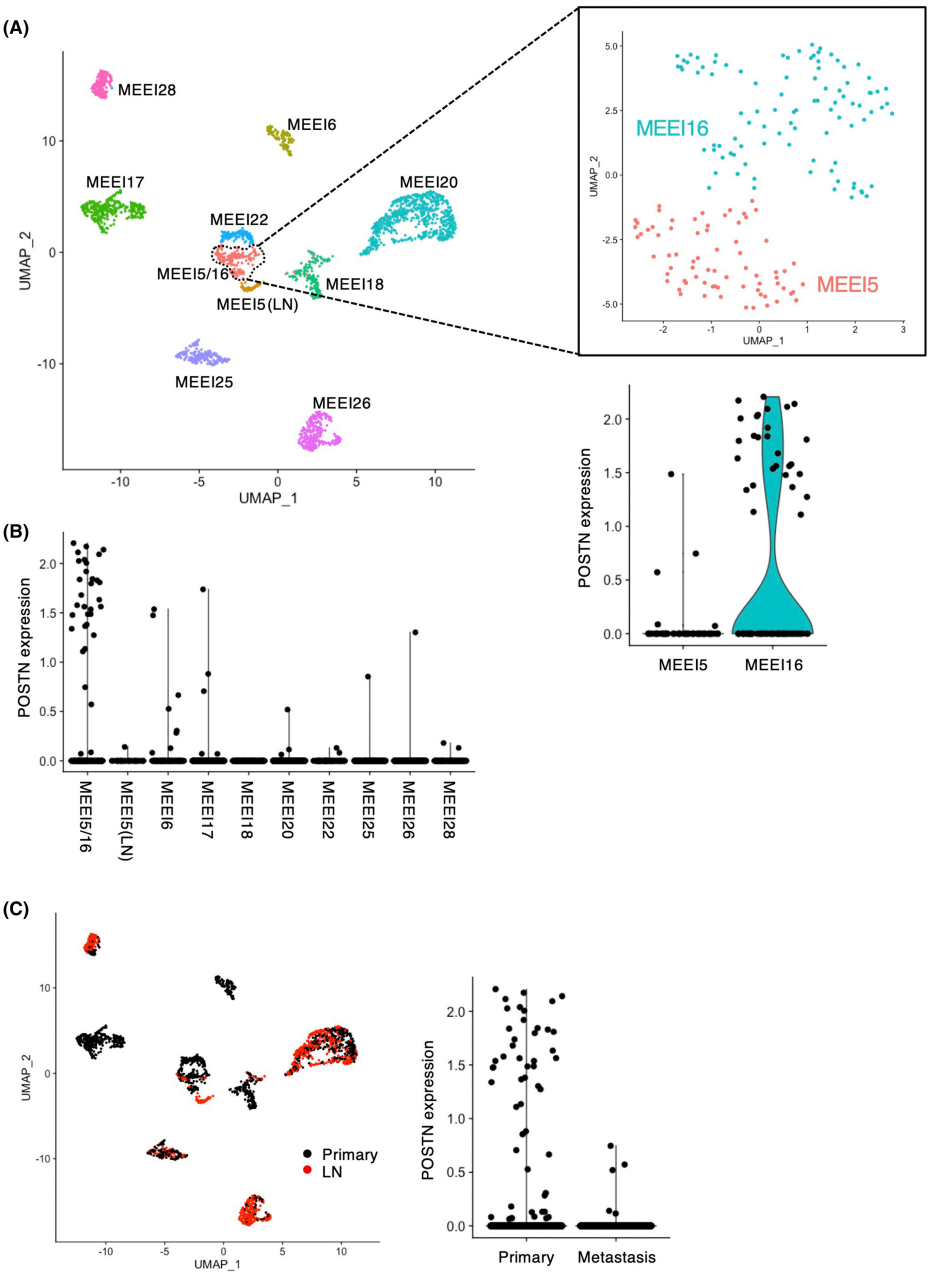
*POSTN* expression in the heterogeneity of cancer cells within the HNSCC tissues. The processed RNA‐seq data obtained from the GEO database (accession number: GSE103322) was used.^31^ (A) UMAP plot of cancer cells from 10 patients (indicated by colors) reveals tumor‐specific clusters. Zoomed in UMAP plot of MEEI5/16 with MEEI5 and MEEI16, which can be seen to further divide into two subclusters. Violin plot depicts distributions of the POSTN expression in MEEI5/16 cluster. (B) Cells with *POSTN* expression are plotted in each cluster. (C) Cells from primary tumors and metastatic lymph nodes are colored black and red. Violin plot depicts distributions of the *POSTN* expression in the cells.

Then, the malignant cells were clustered according to their origin (Figure 1A). The cluster composed of two classical subtype tumors (MEEI5 and MEEI16) preferentially expressed *POSTN* (Figure 1A, B). As a result of re‐clustering, *POSTN* was preferentially expressed in MEEI16. Notably, previous report has revealed that MEEI16 showed a highest p‐EMT score among the HNSCC cases,^28^ suggesting that *POSTN* may be involved in p‐EMT program.

### 3.2 Expression of *POSTN* isoforms in HNSCC cell lines

**FIGURE 2:**
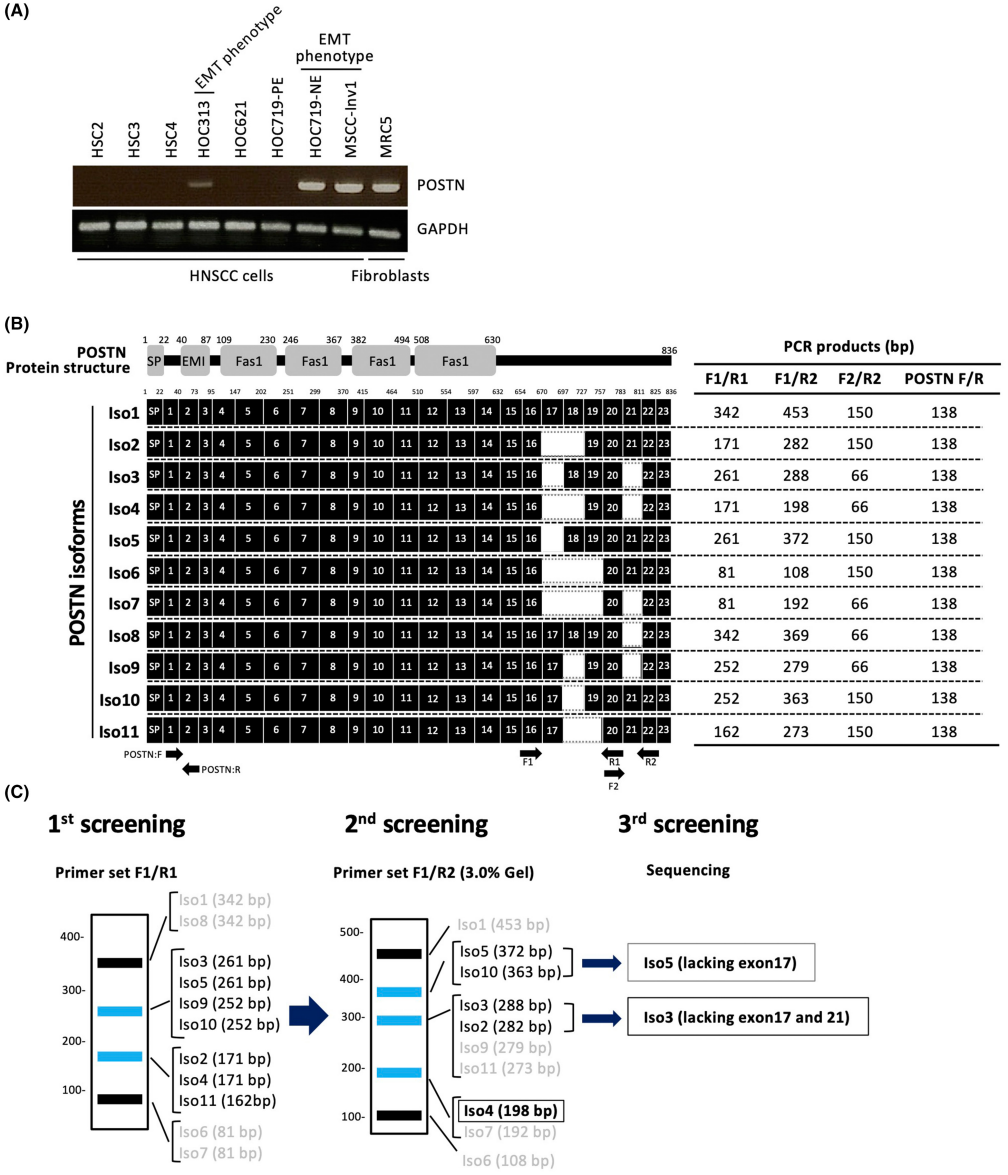
The schematic model of screening methods for *POSTN* isoforms. (A) *POSTN* expression was examined by RT‐PCR in HNSCC cell lines and normal fibroblast. *GAPDH* was used as a control. (B) Periostin protein structure and isoforms (Iso1‐11) are shown. Moreover, primers (F1, F2, R1, and R2) and the product size of each primer set F1/R1, F1/R2 and F2/R2 are shown. (C) The screening method for determination of *POSTN* isoforms using each primer sets.

In HNSCC cell lines and fibroblasts, the *POSTN* expression was examined. Our previous reports showed that HOC313, HOC719‐NE, and MSCC‐Inv1 cells have an EMT phenotype.^39^ *POSTN* expression was detected only in HNSCC cells with an EMT phenotype (HOC313, HOC719‐NE, and MSCC‐Inv1) and fibroblasts (MRC5) (Figure 2A). As HOC719‐NE, MSCC‐inv1 and MRC5 cells showed higher level of *POSTN* expression, we examined the isoforms of *POSTN* in these cells.

POSTN protein contains a secretory signal peptide (SP), a cysteine‐rich EMI domain, and four internal FAS1 repeats (Figure 2B). Human *POSTN* gene exists on chromosome 13, with genes consisting of 23 exons. *POSTN* splicing variants are found at the C‐terminal side between exons 17 and 21. In this study, we examined the detection of all 11 isoforms in MSCC‐Inv1, HOC719‐NE, and MRC5. To screen for *POSTN* isoforms, we designed specific primer sets (F1/R1, F1/R2, and F2/R2) (Figure 2B). These primer sets recognized several isoforms and the expression of *POSTN* isoforms can be detected by combination of RT‐PCR analysis by using these primer sets (Figure 2B, C). Primer set F1/R1 recognized four bands: Iso1/Iso8, Iso3/Iso5/Ios9/Iso10, Iso2/Iso4/Iso11, and Iso6/Iso7 (Figure 2C, left panel). Primer set F1/R2 recognized five bands: Iso1, Iso5/Iso10, Iso2/Iso3, Iso4/Iso7, and Iso6 (Figure 2C, middle panel). Finally, we distinguished the isoforms lacking exon 17 and lacking exon 17 plus exon 21 by sequencing of gel‐extracted PCR products (Figure 2C, right panel).

**FIGURE 3:**
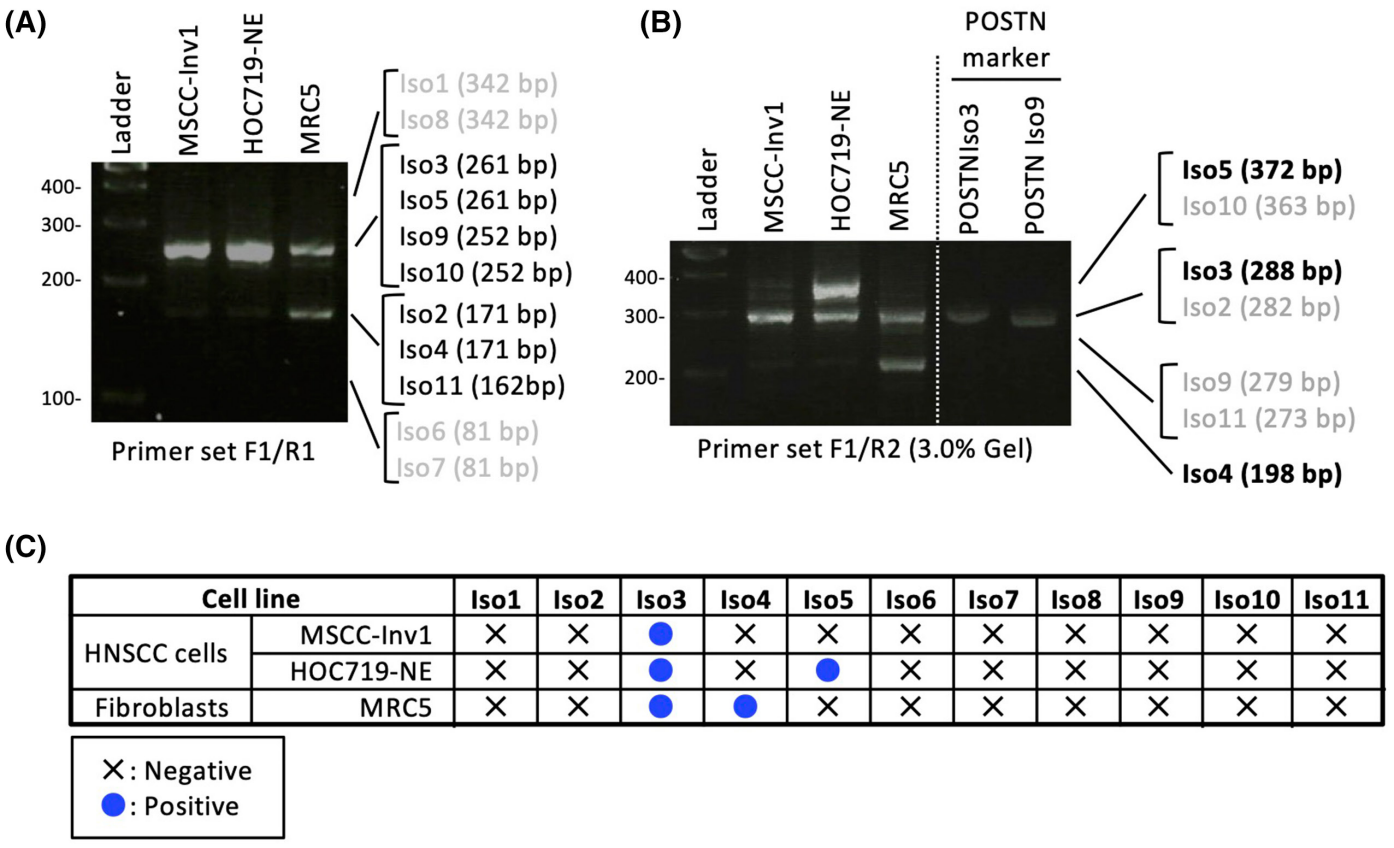
Expression of POSTN isoforms in HNSCC cell lines. (A) *POSTN* isoforms were detected by RT‐PCR using primer set F1/R1 in MSCC‐Inv1, HOC719‐NE, and MRC5 cells. (B) *POSTN* isoforms were detected by RT‐PCR using primer set F1/R2 in MSCC‐Inv1, HOC719‐NE, and MRC5 cells. To distinguish Iso3 and Iso9, PCR products by using primer set F1/R2 from *POSTN* Iso3 and Iso9 constructs were used as a marker. (D) Summary of the expression of *POSTN* isoforms in MSCC‐Inv1, HOC719‐NE, and MRC5 cells.

In all cells, primer set F1/R1 recognized one band: Iso3/Iso5/Ios9/Iso10 in HOC719‐NE and MSCC‐Inv1 cells and two bands: Iso3/Iso5/Ios9/Iso10 and Iso2/Iso4/Iso11 in MRC5 cells (Figure 3A). Then, primer set F1/R2 recognized Iso3 in MSCC‐Inv1 cells, two bands: Iso5/Iso10 and Iso3 in HOC719‐NE cells, and two bands: Iso3 and Iso4 in MRC5 cells (Figure 3B). Iso3 and Iso9/Iso11 could be distinguished by loading PCR products in 3.0% agarose gel. Finally, we performed sequencing the bands for confirming the isoforms. Collectively, MSCC‐Inv1, HOC719‐NE, and MRC5 cells expressed Iso3, Iso4, or Iso5 (Figure 3C). All cells showed strongly positive for Iso3. Interestingly, HOC719‐NE cells showed strongly positive for Iso5, and MRC5 cells showed strongly positive for Iso4. These findings indicated that Iso3 may be a common in HNSCC cells and fibroblasts, but Iso5 may be a specific isoform in some HNSCC cells. Indeed, previous study showed that Iso3 are common in various types of the cells and Iso4 are frequently observed in periodontal ligament cells^41^ (Table 1). However, only one report showed that Iso5 is detected in thyroid tissue and periodontal ligament.^40^

**TABLE 1:** Overview of the POSTN isoforms

In this study POSTN Isoforms	NCBI reference sequence	Exons missing	NCBI	Morra et al., 2011^44^				Morra et al., 2012^45^				Bai et al., 2010^42^	Yamada et al., 2014^41^	Kim et al., 2008^46^
					RCC	Normal kidney	Fetal Kidney		NSCLC	Normal lung	Fetal lung	Thyroid	Periodontal ligament	TCC
Iso1	NP_006466.2	None	Iso1	Iso1	−	−	+	Iso1	−	−	+	thy1	Type I	WT
Iso2	NP_001129406.1	17, 18	Iso2	Iso2	−	−	+	Iso2	+	+	+	thy3	−	Variant III
Iso3	NP_001129407.1	17, 21	Iso3	Iso3	+	+	+	Iso3	+	+	+	thy6	Type III	Variant II
Iso4	NP_001129408.1	17, 18, 21	Iso4	Iso4	+	+	+	Iso4	+	+	+	thy7	Type II	Variant I
Iso5	NP_001273594.1	17		−	ND	ND	ND	−	ND	ND	ND	thy2	Type V	−
Iso6	NP_001273595.1	17, 18, 19	Iso6	Iso7	−	−	+	Iso7	+	+	+	thy4	−	−
Iso7	NP_001273596.1	17, 18, 19, 21	Iso7	Iso8	+	+	+	Iso8	+	+	+	thy8	−	−
Iso8	NP_001317446.1	21	Iso8	Iso5	−	−	+	Iso5	−	−	+	thy5	−	−
Iso9	−	18, 21	−	−	−	−	+	Iso9	−	−	+	−	−	−
Iso10	−	18	−	Iso6	−	−	+	Iso6	−	−	−	−	−	−
Iso11	−	18, 19	−	−	ND	ND	ND	−	ND	ND	ND	−	Type IV	−

### 3.3 Induction of *POSTN* isoforms by TGF‐β1

**FIGURE 4:**
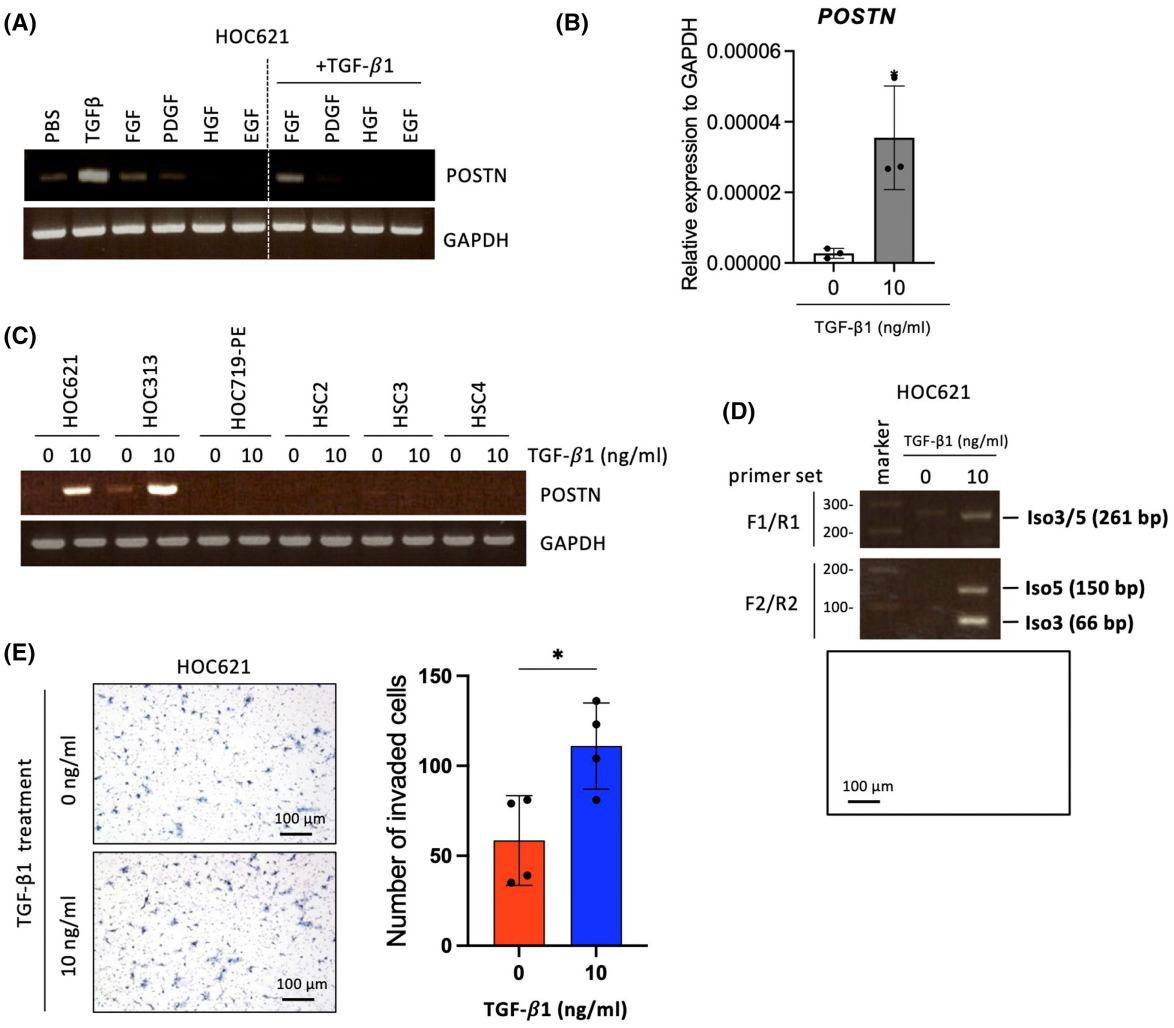
The upregulation of *POSTN* expression and detection of isoforms in TGF‐β1 treated HNSCC cell lines. (A) HOC621 cells were treated with TGF‐β1, FGF2, PDGF, HGF, and EGF for 24 hours. Cells were then collected and analyzed *POSTN* expression by RT‐PCR. GAPDH was used as a control. (B) *POSTN* mRNA expression was determined by qRT‐PCR in HOC621 cells after TGF‐β1 treatment. In each group, data were presented as the mean ± SD of triplicates. *P < 0.05 (C) HNSCC cell lines (HOC621, HOC313, HOC719‐NE, HSC2, HSC3, and HSC4) were treated with 10 ng/mL of TGF‐β1 for 24 hours. Cells were then collected and analyzed *POSTN* expression by RT‐PCR. *GAPDH* was used as a control. (D) *POSTN* isoforms were detected by RT‐PCR using primer set F1/R1 or F2/R2 in TGF‐β1 treated HOC621 cells. (E) The invasion ability of HOC621 cells with or without TGF‐β1 treatment was examined by in vitro invasion assay. Representative images were shown. Graph shows the number of invaded cells as mean ± SEM (*n* = 3). *p < 0.05.

Previous report showed that several growth factors including TGF‐β1, BMP, and VEGF induced *POSTN* expression in a cell‐specific context.^7^ In HNSCC tissue, TGF‐β1 was produced by several types of the cells (Figure S2A) and its receptor, TGFBR2 as well as *POSTN* was preferentially expressed in tumors including where p‐EMT program was detected (MEEI5, 16, 17, and 28) (Figure S2B). Then, we treated growth factors including TGF‐β1, FGF2, PDGF, HGF, and EGF in HOC621 to examine the induction of *POSTN*. Among growth factors, TGF‐β1 markedly induced *POSTN* expression (Figure 4A, B). We examined *POSTN* induction by TGF‐β1 in HNSCC cells with low *POSTN* expression or without *POSTN* expression. *POSTN* was induced by TGF‐β1 treatment in HOC621 and HOC313 cells (Figure 4C). In HOC621 cells, both *POSTN* Iso3 and Iso5 were induced by TGF‐β1 treatment (Figure 4D). Indeed, the invasion of HOC621 cells was significantly promoted by TGF‐β1 treatment (Figure 4E).

### 3.4 Synergistic effect of *POSTN* Iso5 for promoting invasion by Iso3

Screening for *POSTN* isoforms by combination of several primer sets revealed that HNSCC cells expressed *POSTN* Iso3 and Iso5 as shown in Figure 3C. Iso3 is known as a common isoform in various types of cancer. Indeed, we previously showed that Iso3 promoted invasion and metastasis in HNSCC.^4^, ^9^ However, the role of Iso5 remains unclear. Therefore, here we focused on the role of Iso5 in HNSCC.

**FIGURE 5:**
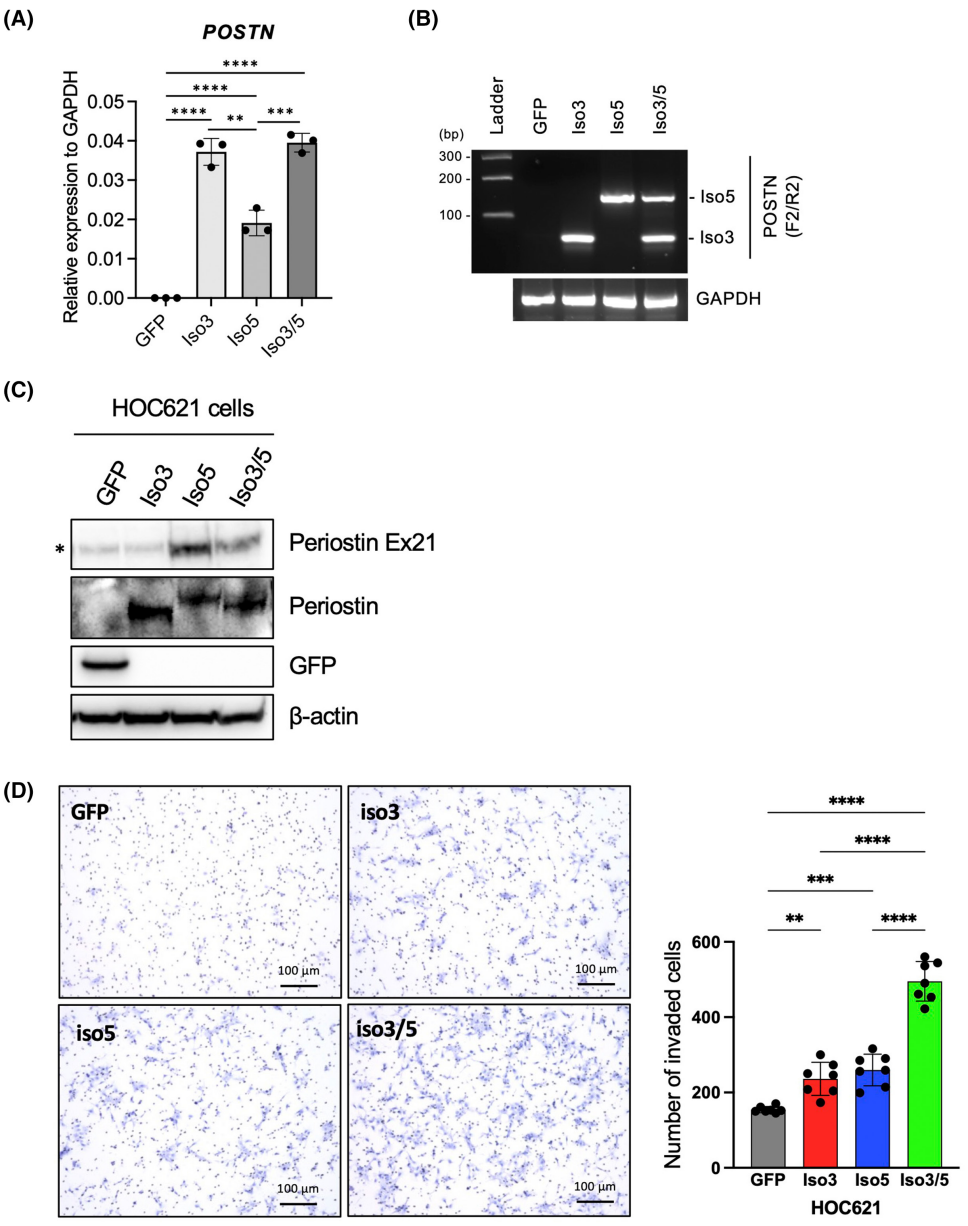
*POSTN* Iso5 cooperatively promotes invasion with Iso3 which is conventional isoform. (A) HOC621 cells were engineered to overexpressing GFP, *POSTN* Iso3 and/or Iso5 by transfection with lentiviral gene transfer. The overexpression of GFP was used as a control. Ectopic expression of *POSTN* Iso3 and Iso5 was quantified by qRT‐PCR. In each group, data are presented as the mean ± SD of triplicates. ****p < 0.001, ***p < 0.005, **p < 0.01. (B) Ectopic expression of *POSTN* Iso3 and Iso5 were detected by RT‐PCR using primer set F2/R2. GAPDH was used as a loading control. (C) Ectopic expression of Periostin Iso3 and Iso5 with monensin treatment was examined by immunoblotting with antibodies detecting N‐terminus and exon 21 of *POSTN*. anti‐Ex21 periostin antibody can detect *POSTN* Iso5 but not Iso3 lacking Exon 21. β‐Actin was used as a control. (D) The invasion ability of GFP‐, *POSTN* Iso3‐, Iso5, and Iso3/5‐overexpressing HOC621 cells were examined by in vitro invasion assay. Representative images were shown. Graph shows the number of invaded cells as mean ± SEM (*n* = 3). ****p < 0.001, ***p < 0.005, **p < 0.01.

We generated Iso5‐overexpressing cells by using HOC621 cells without *POSTN* expression (Figure 5A, B). We also generated empty vector (GFP)‐transfected cells and Iso3‐overexpressing cells. Ectopic expression of *POSTN* Iso3 and Iso5 did not change the morphology and cell proliferation, compared with GFP (data not shown). We also detected periostin isoforms after treatment with monensin, which blocks the transport from the medial to the trans cisternae of the Golgi stack (Figure 5C). Anti‐periostin antibody recognized both Iso3 and Iso5, anti‐periostin Ex21 antibody recognized Iso5 (Figure 5C).

By using Iso5‐overexpressing cells, we compared the invasion ability with control (GFP‐transfected cells) and Iso‐3 overexpressing cells. Iso5 as well as Iso3 promoted invasion at the similar level, in comparison with GFP‐transfected cells (Figure 5D). Moreover, we examined the synergistic effect of Iso3 and Iso5 (Iso3/5) in the invasion. Interestingly, Iso3/5 overexpression remarkably promoted invasion comparing with single overexpression, even though the total expression of *POSTN* was comparable level (Figure 5A–D). Thus, *POSTN* Iso5 promoted the invasion and co‐expression with Iso3 showed synergistic effect for invasion.

### 3.5 Suppressed invasion by POSTN Iso5 depletion

**FIGURE 6:**
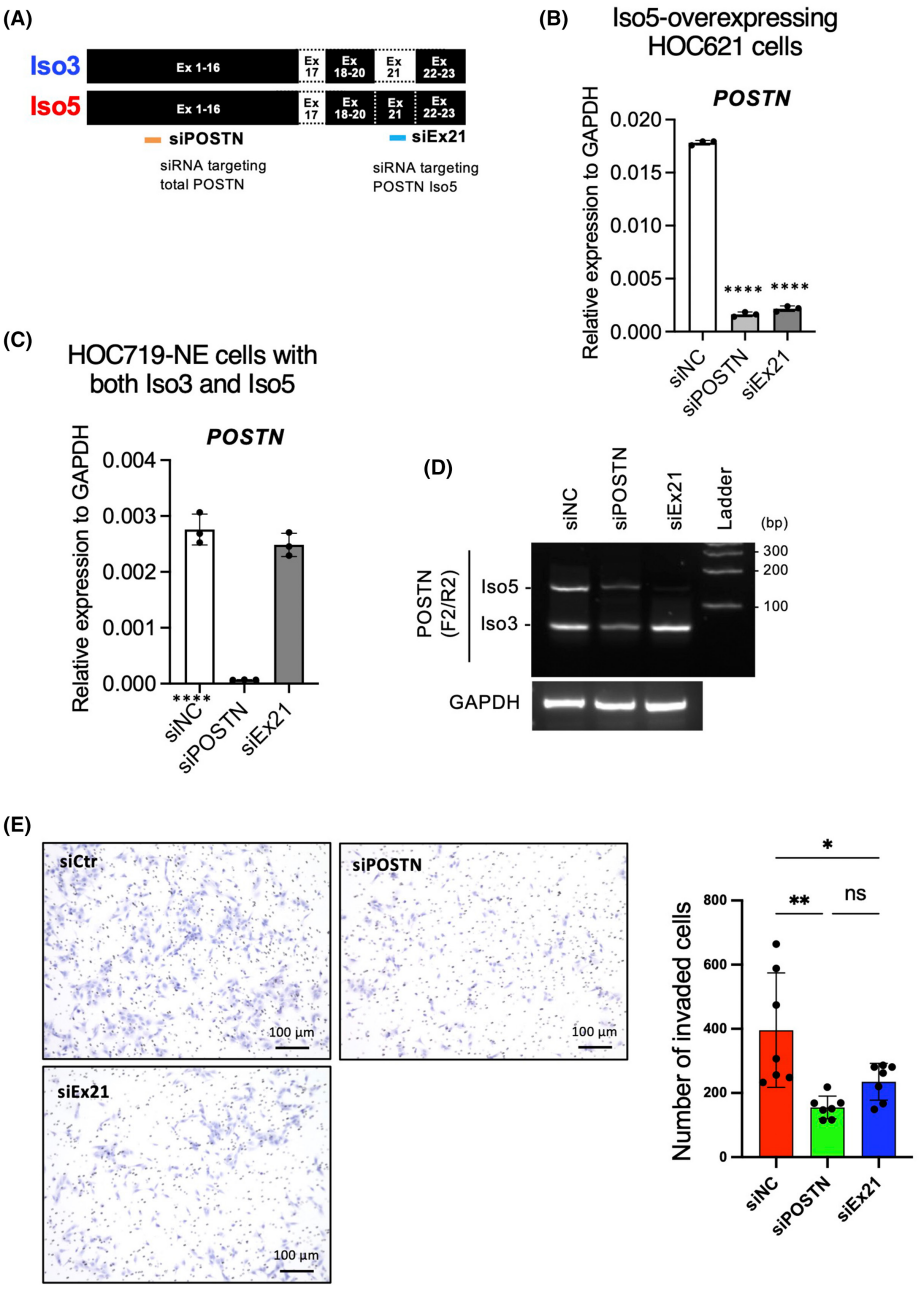
Knockdown of *POSTN* Iso5 attenuates invasion ability in HNSCC cell line having EMT feature. (A) The structure of *POSTN* Iso3 and Iso5 are shown. Moreover, siRNA targeting *POSTN* Iso5 (siEx21) or total *POSTN* are shown. (B) *POSTN* mRNA expression in Iso5‐overexpressing HOC621 cells were determined by qRT‐PCR. In each group, data are presented as the mean ± SD of triplicates. ****p < 0.001 (C) *POSTN* mRNA expression in negative control siRNA (siNC), total *POSTN* siRNA (siPOSTN), and siRNA targeting *POSTN* Iso5 (siEx21)‐transfected HOC719‐NE cells were determined by qRT‐PCR. In each group, data are presented as the mean ± SD of triplicates. ****p < 0.001 (D) *POSTN* isoforms were detected by RT‐PCR using primer set F2/R2 in siNC, siPOSTN, and siEx21‐transfected HOC719‐NE cells. *GAPDH* was used as a control. (E) The invasion ability of siNC and siEx21‐transfected HOC719‐NE cells were determined by in vitro invasion assay. Representative images were shown. Graph shows the number of invaded cells as mean ± SEM (*n* = 3). ****p < 0.001.

To confirm the role of *POSTN* Iso5 in the invasion, we designed siRNA (siEx21) targeting exon 21 for the specific knockdown of Iso5 (Figure 6A). In addition, we designed siRNA (siPOSTN) targeting all isoforms of *POSTN* (Figure 6A). We confirmed the knockdown efficiency after transfection of these siRNAs in HOC621 cells overexpressing with *POSTN* Iso5. *POSTN* Iso5 expression was significantly downregulated by both siPOSTN and siEx21 siRNAs (Figure 6B). Next, we transfected these siRNAs to HOC719‐NE cells with the expression of both *POSTN* Iso3 and 5. The total expression of *POSTN* including Iso3 and Iso5 was suppressed by siPOSTN (Figure 6C, D). On the other hand, siEx21 specifically suppressed the expression of Iso5 but not Iso3. Then, we performed in vitro invasion assay after depletion of Iso5 by using siEx21 in HOC719‐NE cells. Depletion of Iso5 did not affect the morphology and slightly decreased the cell growth (data not shown). In HOC719‐NE cells, Iso5 depletion remarkably suppressed the invasion without affecting total *POSTN* levels (Figure 6C, E), indicating that *POSTN* promoted the invasion and that co‐expression with Iso3 and 5 showed synergistic effect for invasion.

### 3.6 Upregulation of p‐EMT genes by POSTN Iso3 and Iso5 overexpression

**FIGURE 7:**
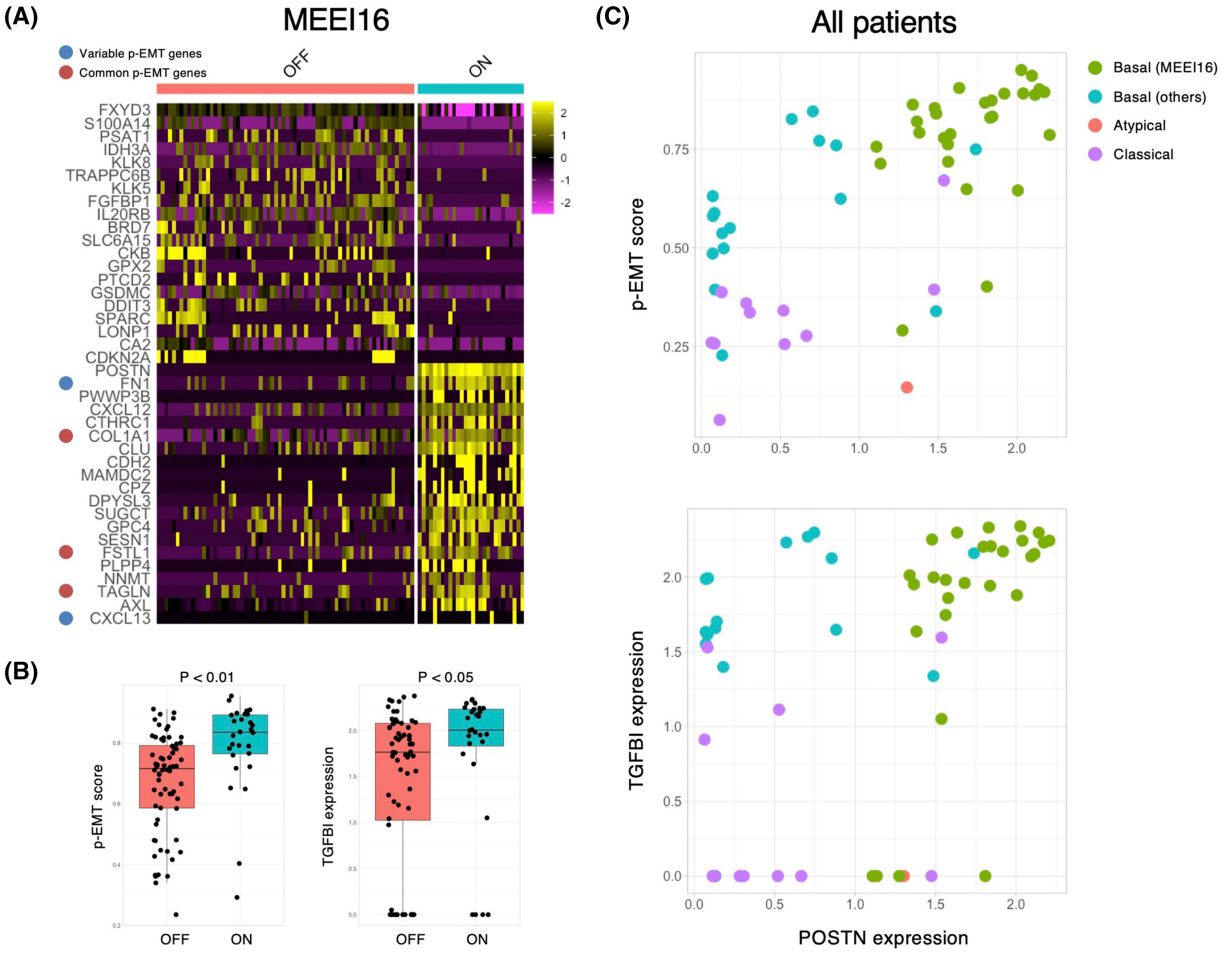
Correlation between *POSTN* and p‐EMT. (A) Heatmap shows top20 genes (columns) that are differentially expressed across *POSTN* expressing cells (ON) and *POSTN* nonexpressing cells (OFF) in MEEI16 tumor. (B) Boxplot shows p‐EMT score and *TGFBI* expression values in ON and OFF cells. (C) Scatter plot of p‐EMT score and *POSTN* expression value (upper), *TGFBI* expression value and *POSTN* expression value (lower) for ON cells in all tumors were shown. Each cell was colored by the reported TCGA molecular subtype.

As shown in Figure 2A, *POSTN* expression was observed only in HNSCC cells with EMT features. Moreover, POSTN was preferentially expressed in MEEI16 with highest p‐EMT score among the HNSCC cases (Figure 1A),^31^ suggesting that *POSTN* may be involved in p‐EMT program. A previous single‐cell analysis using HNSCC tissues identifies p‐EMT‐related genes.^31^ By using this data, we compared *POSTN* expression with p‐EMT‐related genes. Interestingly, cells expressing *POSTN* transcripts (ON) in MEEI16 tumor expressed multiple common and variable p‐EMT genes at higher levels compared to cells not expressing *POSTN* (OFF) (Figure 7A). ON cells showed higher p‐EMT scores and *TGFBI* expression, a representative p‐EMT gene and homologous to POSTN (Figure 7B). Moreover, the correlation between *POSTN* expression and p‐EMT score in all type of tumors was examined. *POSTN* expression was strongly correlated with p‐EMT score in basal tumors where the p‐EMT program was detected, while both *POSTN* expression and p‐EMT score were lower in classical and atypical tumors^31^ (Figure 7C). There were no differences in *TGFBR* expression between MEEI16 and other tumors, and between ON and OFF cells, suggesting the existence of a p‐EMT induction mechanism specific to *POSTN*‐expressing cells (Figure S2B, C).

**FIGURE 8:**
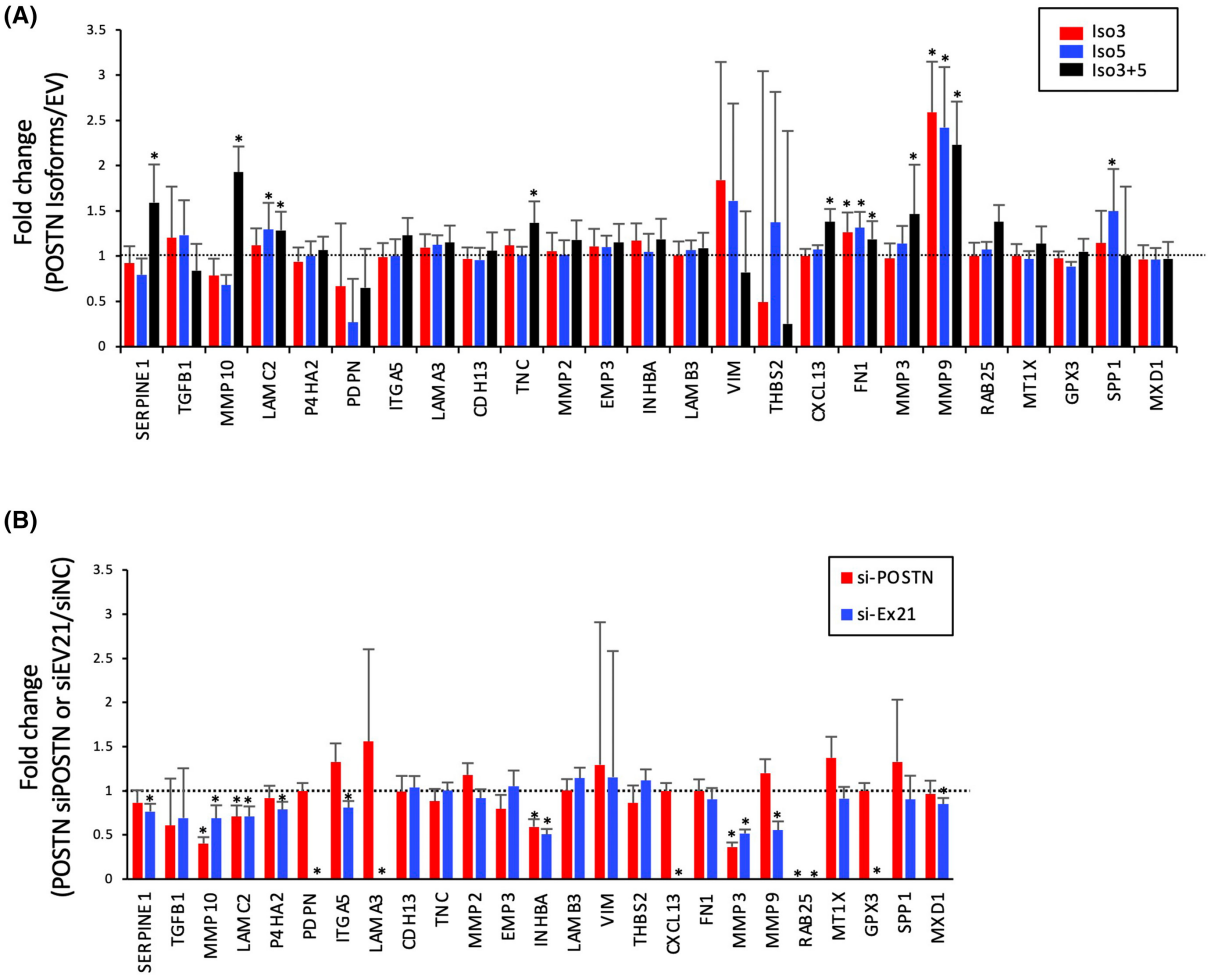
Induction of p‐EMT genes by *POSTN* Iso3 and/or Iso5. (A) HOC621 cells were engineered to overexpressing *POSTN* Iso3 and/or Iso5 by transfection with lentiviral vector. The overexpression of GFP was used as a control. Expression of p‐EMT genes was evaluated by qPCR in EV‐, *POSTN* Iso3‐, Iso5, and Iso3/5‐transfected HOC621 cells. The graphs show the results as mean ± S.D. of three independent experiments. *p < 0.05 (compared to EV). (B) Expression of p‐EMT genes was evaluated by qPCR in negative control siRNA (siNC), siRNA targeting *POSTN* Iso3 and 5 (siPOSTN), and siRNA targeting *POSTN* Iso5 (siEx21)‐transfected HOC719‐NE cells with both *POSTN* Iso3 and Iso5 expression. The graphs show the results as mean ± S.D. of three independent experiments. *p < 0.05 (compared to siNC).

To know the involvement of *POSTN* in p‐EMT program, we investigated the expression of p‐EMT‐related genes in Iso3‐, Iso5‐, and Iso3/5‐overexpressing HOC621 cells by qPCR and compared with GFP‐transfected control cells. Upregulation of several p‐EMT‐related genes were observed in *POSTN* Iso3‐, Iso5‐, and Iso3/5‐overexpressing cells, in comparison with control cells (Figure 8A). Moreover, we examined the effect of siPOSTN (targeting both Iso3 and Iso5) and siEx21 (targeting Iso5) on the expression of p‐EMT‐related genes in HOC719‐NE cells with Iso3 and Iso5 expression. Several p‐EMT‐related genes were downregulated by siPOSTN or siEx21 (Figure 8B). In POSTN Iso3/5 overexpressing cells, SERPINE1, MMP10, TNC, CXCL13, and MMP3 were upregulated (Figure 8A). Moreover, among them, siEX21 and siPOSTN downregulated MMP10, LAMC2, INHBA, MMP3, and RAB25 (Figure 8B). Therefore, MMP10 and MMP3 may be involved in p‐EMT induction by POSTN isoforms (Iso3 and Iso5).

## 4 DISCUSSION

It has been widely accepted that *POSTN* is closely involved in cancer progression.^35^ In this study, *POSTN* expression is preferentially observed in endothelial cells, CAFs, and HNSCC cells with the highest p‐EMT score. Indeed, cells with p‐EMT phenotype localize close to CAFs at the leading edge of primary tumor tissues.^31^ Moreover, a TGF‐β‐responsive gene, TGFBI is one of the top‐scoring genes in the p‐EMT program.^31^ Interestingly, POSTN is highly homologous to TGFBI with FAS1 domains.^41^ Indeed, TGF‐β1 upregulates *POSTN* in certain type of HNSCC cells (Figure 4C). From these observations, *POSTN* may be concerned with the p‐EMT program. It is well accepted that cancer cells with p‐EMT phenotype show a higher metastatic property, compared with complete EMT phenotype. Interestingly, overexpression of *POSTN* Iso3 and/or five upregulated p‐EMT‐related genes. As Iso5 depletion suppressed several p‐EMT‐related genes, Iso5 may be contributed to p‐EMT program. Although *POSTN* itself could not induce EMT evaluated by morphology and loss of E‐cadherin (data not shown), *POSTN* may promote tumor progression via p‐EMT program under TGF‐β signaling pathway.

Recently, it has been revealed that *POSTN* has several different splicing variants.^7^, ^32^, ^33^ However, the expression pattern of *POSTN* isoforms in HNSCC and the functional difference among *POSTN* isoforms are not fully understood. To date, eight different isoforms of *POSTN* are registered in NCBI (Table 1). In addition, other three isoforms were also reported in various types of tissues.^40^, ^42^, ^43^, ^44^, ^45^ *POSTN* isoforms differ in their C‐terminal sequences (exons 17–21). On the other hand, N‐terminal sequences are common, and the EMI and Fas1 domains within N‐terminus are responsible for integrin binding^37^ (Figure 2B). In previous studies, *POSTN* isoforms were examined by PCR in the various tissues.^40^, ^42^, ^43^, ^44^, ^45^ However, several types of the cells are included in the tissues. Therefore, here we used the cell lines to know the expression pattern of *POSTN* isoforms. Indeed, *POSTN* expression was only observed in HNSCC cells with EMT features. These HNSCC cells and fibroblasts expressed Iso3 and 4. Iso3 was a common isoform in HNSCC cells and fibroblasts and construct of Iso3 has been used for overexpression experiments in previous studies.^40^, ^42^, ^43^, ^44^, ^45^ Iso4 is also frequently observed in various types of the tissue.^40^, ^42^, ^43^, ^44^, ^45^ However, only so far has been reported that Iso5 is detected in fetal kidney.^43^ This is the first report that Iso5 is detected in cancer cells. We think that Iso5 may be cancer cell‐derived specific isoform in HNSCC cells.

The phenotypes driven by *POSTN* overexpression may be caused by promoted the downstream signals including migration, invasion, and survival via acting as a ligand for integrins.^4^, ^10^, ^11^, ^46^, ^47^ In this study, we found co‐expression of Iso3 and Iso5 in HNSCC cells with EMT feature and in TGF‐β1 treated HNSCC cells. Interestingly, invasion ability driven by co‐expression of Iso3 and Iso5 was higher than that by single overexpression. As shown in Figure 2B, Iso3 lacks exon 17 and 21, and Iso5 lacks exon 17. Therefore, exon 21 may have synergistic effect with Iso3. *POSTN* activated several signaling pathways such as Akt/PKB pathway, YAP/TAZ pathway via the αvβ3 integrin‐FAK/Src axis.^10^, ^48^ *POSTN* expressing cells in MEEI16 tumors highly expressed the αvβ3 integrin ligands, such as *FN1* and *CXCL12* and p‐EMT genes.^49^, ^50^ The αvβ3 integrin signaling has recently been reported to induce p‐EMT, and signals from these integrin ligands, including *POSTN*, may contribute to the promotion of p‐EMT program.^51^ Hence, it is interesting issue to analyze αvβ3 integrin‐FAK/Src signaling by co‐treatment of Iso3 and Iso5. In the follow‐up studies, we will examine the expression of POSTN Iso5 in a larger number of HNSCC cases by using a specific antibody against exon 21 generated in this study. During EMT, ESRP1 and ESRP2 (Epithelial Splicing Regulatory Proteins 1 and 2) coordinate an epithelial cell‐type‐specific splicing program via the alternative splicing of FGFR‐encoding transcripts.^52^, ^53^ Therefore, the change in alternative splicing may be essential for p‐EMT program.

This is the first report of cancer cell‐specific splicing variant of *POSTN*. However, as a limitation of this study, it is difficult to examine the expression of *POSTN* isoforms in HNSCC tissues. Previously, gene function analysis in TCGA bulk RNA‐seq data using various bioinformatics approaches has been reported.^54^, ^55^ In the case of *POSTN*, such analysis using bulk RNA‐seq data is difficult, because multiple cell types express multiple POSTN isoforms. To solve this problem, we should develop in situ hybridization by using a specific probe of each *POSTN* isoform. In this study, we identified a *POSTN* isoform lacking exon 17 (Iso5) as a promoting factor of tumor progression via p‐EMT program in HNSCC. HNSCC cells having co‐expression of *POSTN* Iso3 and Iso5 may possess more aggressive invasive potential. We also suggest that *POSTN* Iso5 can be a useful marker for detecting cancer cells undergoing EMT. Moreover, detection of *POSTN* Iso5 can be a novel diagnostic marker and therapeutic target in HNSCC.

## AUTHOR CONTRIBUTIONS

**Shao Wenhua:** Data curation (equal); investigation (equal); writing – original draft (equal). **Takaaki Tsunematsu:** Conceptualization (equal); data curation (equal); funding acquisition (equal); investigation (equal); methodology (equal); resources (equal); visualization (equal); writing – original draft (equal). **Masaki Umeda:** Investigation (equal). **Hiroaki Tawara:** Investigation (supporting). **Natsumi Fujiwara:** Data curation (equal); formal analysis (equal); investigation (supporting); methodology (equal). **Yasuhiro Mouri:** Data curation (equal); formal analysis (equal); investigation (equal); methodology (equal); validation (equal). **Rieko Arakaki:** Investigation (supporting); methodology (supporting). **Naozumi Ishimaru:** Methodology (supporting); supervision (supporting); writing – review and editing (supporting). **Yasusei Kudo:** Conceptualization (lead); data curation (equal); formal analysis (equal); funding acquisition (lead); project administration (lead); resources (lead); supervision (lead); validation (lead); visualization (lead); writing – original draft (equal); writing – review and editing (lead).

## FUNDING INFORMATION

This work was supported by grants to Y. Kudo from JSPS KAKENHI [22K19629, 22H03288, and 21KK0162]. This work was also supported by grants to T. Tsunematsu from JSPS KAKENHI [20K18480] and Nishiyama Dental Academy Foundation.

## CONFLICT OF INTEREST

The authors have no conflict of interest.

## Supporting information


Figure S1–S2.
