# Supplementary material for: Cancer cell‐derived novel periostin isoform promotes invasion in head and neck squamous cell carcinoma
Source: Cancer Med. 2023 Jan 23;12(7):8510–25. doi: 10.1002/cam4.5601 (PMC10134278; doi:10.1002/cam4.5601)
Supplement: Supplementary file 1 — Figure S1–S2. [file CAM4-12-8510-s001.pdf]

Supplementary information

Cancer cell-derived novel periostin isoform promotes invasion in head and neck squamous cell carcinoma

Shao Wenhua, Takaaki Tsunematsu, Masaaki Umeda, Natsumi Fujiwara, Yasuhiro Mouri, Rieko Arakaki, Naozumi Ishimaru, Yasusei Kudo

Supplementary Figure S1

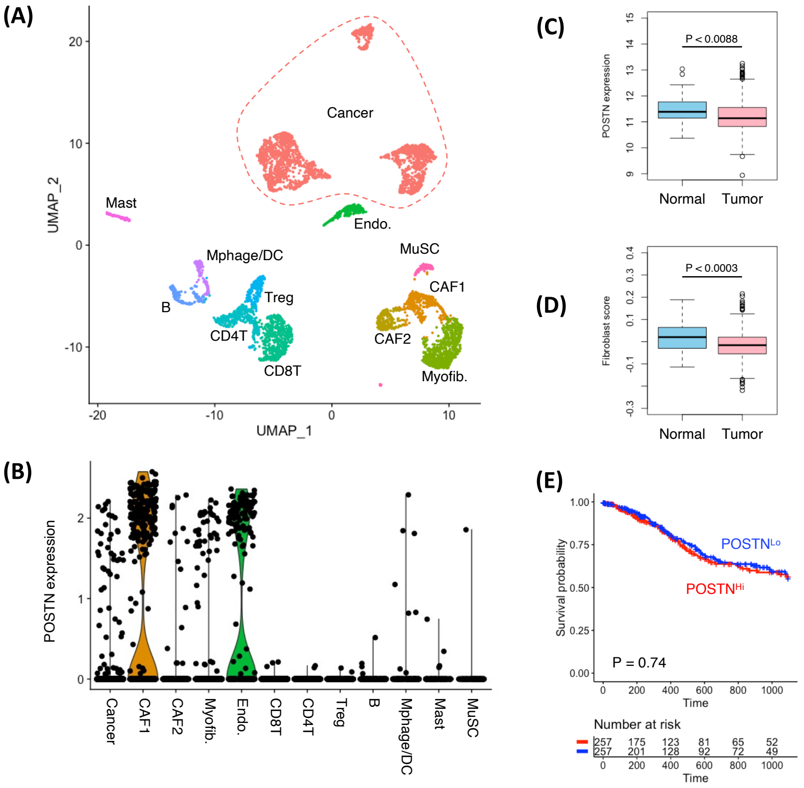

Supplementary Figure S1. POSTN expression in the heterogeneity of malignant and non-malignant cells within the HNSCC tissues.

The processed scRNAseq data obtained from the Gene Expression Omnibus (GEO) database, (accession number: GSE103322), was used[31]. (A) UMAP clustering of all cells including malignant and non-malignant cells was shown. (B) Violin plot depicts distributions of the *POSTN* expression in malignant and non-malignant cells. (C) The comparison of *POSTN* expression between normal and tumor tissues in TCGA-HNSC. (D) The comparison of fibroblast abundance between normal and tumor tissues in TCGA-HNSC. (E) The three-year overall survival in TCGA-HNSC. HNSC patients were divided by the median POSTN expression.

Supplementary Figure S2

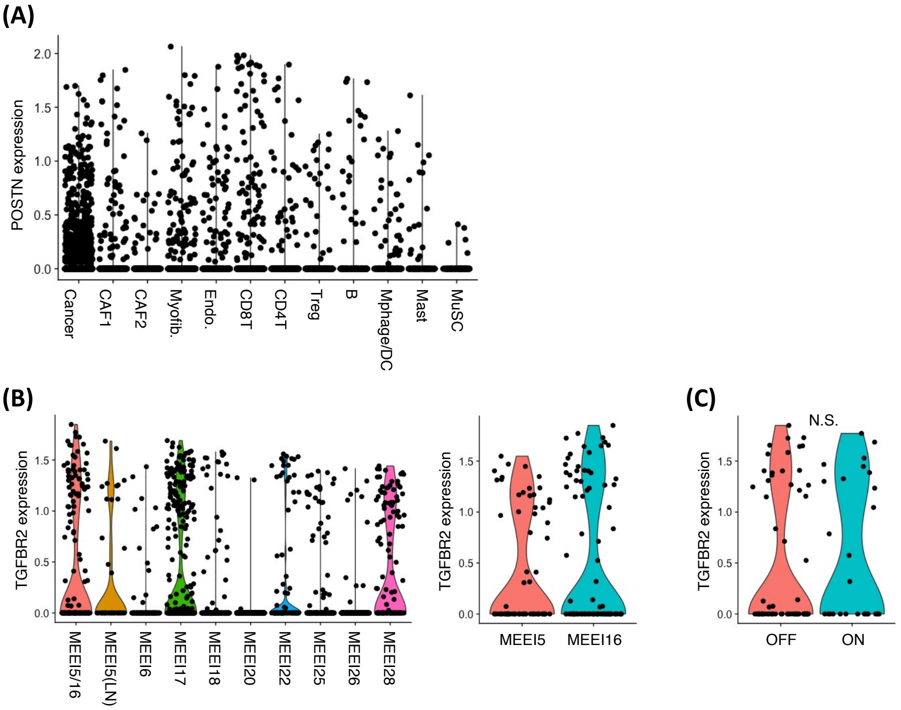

Supplementary Figure S2. TGFB1 and TGFBR2 expression in the heterogeneity of non-malignant cells and cancer cells within the HNSCC tissues.

The processed sc-RNAseq data obtained from the Gene Expression Omnibus (GEO) database, (accession number: GSE103322), was used[31]. (A) Violin plot depicts distributions of the TGFB1 expression in all cell types. (B) Violin plot depicts distributions of the TGFBR2 expression in malignant cells. (C) Violin plot depicts distributions of the *TGFBR2* expression in *POSTN* expressing and non-expressing malignant cells in MEEI16 tumor.
